# Mesenchymal Stem/Stromal Cell-Derived Exosomes for Immunomodulatory Therapeutics and Skin Regeneration

**DOI:** 10.3390/cells9051157

**Published:** 2020-05-07

**Authors:** Dae Hyun Ha, Hyun-keun Kim, Joon Lee, Hyuck Hoon Kwon, Gyeong-Hun Park, Steve Hoseong Yang, Jae Yoon Jung, Hosung Choi, Jun Ho Lee, Sumi Sung, Yong Weon Yi, Byong Seung Cho

**Affiliations:** 1 ExoCoBio Exosome Institute (EEI), ExoCoBio Inc., Seoul 08594, Korea; dh.ha@exocobio.com (D.H.H.); hyunkeun.kim@exocobio.com (H.-k.K.); junho.lee@exocobio.com (J.H.L.); sumi.sung@exocobio.com (S.S.); 2 School of Chemical and Biological Engineering, Seoul National University, Seoul 08826, Korea; purequill@naver.com; 3 Oaro Dermatology Clinic, Seoul 13620, Korea; banbury@hanmail.net; 4 Department of Dermatology, Dongtan Sacred Heart Hospital, Hallym University College of Medicine, Hwasweong-si, Gyeonggi-do 18450, Korea; jin66666@hanmail.net; 5 Guam Dermatology Institute, Tamuning, GU 96913, USA; guamderm@gmail.com; 6 Oaro Dermatology Clinic, Seoul 01695, Korea; jaeyoon007@hanmail.net; 7 Piena Clinic, Seoul 06120, Korea; hosungeee@naver.com

**Keywords:** anti-aging, anti-inflammation, hair growth, immunomodulation, mesenchymal stem cells (MSCs), MSC-exosomes, skin barrier, therapeutics, regenerative aesthetics, wound healing

## Abstract

Exosomes are nano-sized vesicles that serve as mediators for cell-to-cell communication. With their unique nucleic acids, proteins, and lipids cargo compositions that reflect the characteristics of producer cells, exosomes can be utilized as cell-free therapeutics. Among exosomes derived from various cellular origins, mesenchymal stem cell-derived exosomes (MSC-exosomes) have gained great attention due to their immunomodulatory and regenerative functions. Indeed, many studies have shown anti-inflammatory, anti-aging and wound healing effects of MSC-exosomes in various in vitro and in vivo models. In addition, recent advances in the field of exosome biology have enabled development of specific guidelines and quality control methods, which will ultimately lead to clinical application of exosomes. This review highlights recent studies that investigate therapeutic potential of MSC-exosomes and relevant mode of actions for skin diseases, as well as quality control measures required for development of exosome-derived therapeutics.

## 1. Introduction

The discovery of extracellular vesicles (EVs) or exosomes goes back to the 1940s, and these tiny vesicles were ignored as cellular garbage bins for a long time [1,2,3]. They only began to draw significant attention around the mid-2000s after re-discovery of exosomes as messengers for cell-to-cell communications [1,4,5,6]. It is no exaggeration to say that we are at the dawn of the exosome era. There were more than three thousand publications on EVs or exosomes and related subjects in PubMed annually in 2018 and 2019 [1]. The race toward commercialization of exosome-based therapeutics has already begun [7,8,9,10]. The top four exosome start-up companies, Codiak Biosciences, Exosome Diagnostics, Evox Therapeutics, and ExoCoBio have received approximately $386.2 million in investor funding [8]. In addition, several big deals have been made between exosome start-ups and big pharma companies [10].

Exosomes are nano-sized extracellular vesicles (EVs) released by almost all eukaryotic cells [11]. In general, their size ranges from 30 nM to 200 nM. Two other subpopulations of EVs are microvesicles (100–1000 nM) and apoptotic bodies (500–2000 nM) [12,13,14]. Exosomes derived from stem cells have attractive therapeutic potential in several aspects [15]. It has been established that the mode of action (MoA) for therapeutic effects of stem cells is mainly paracrine effects mediated by secreted factors from stem cells [6,16]. Among parts of the secretome of stem cells, exosomes have been reported to play the major role in the paracrine effects [16,17,18]. Mesenchymal stem/stromal cells (MSCs) are the most preferable source of therapeutic exosomes, since MSCs themselves appear to be safe based on huge amount of clinical data over the last decade [15]. In addition, MSC-derived exosomes (MSC-exosomes) can be sterilized by filtration and produced as an off-the-shelf product, while MSCs themselves cannot. Moreover, MSC-exosomes are considered to be free from the safety issues in the context of cell-based therapy, such as tumorigenic potential by cell administration [19,20]. Indeed, MSC-exosomes have been applied as alternatives to MSCs for new cell-free therapeutic strategies in a variety of disease models including neurological, cardiovascular, immune, renal, musculoskeletal, liver, respiratory, eye, and skin diseases, as well as cancers [15,17,19,21,22].

## 2. MSCs as Sources of Exosomes

MSCs have both self-renewal capabilities (i.e., they can generate more MSCs themselves) and differentiation (into other types of cells) potentials [23]. MSCs can be obtained from a range of tissues and body fluids, such as adipose tissue, bone marrow (BM), dental pulp, synovial fluid (SF), amniotic fluid (AF), placenta (PL), umbilical cord (UC), umbilical cord blood (UCB), and Wharton’s jelly (WJ) [24]. MSCs can also be derived from embryonic stem cells (ESCs) or induced pluripotent stem cells (iPSCs) [25,26,27]. MSCs, depending on their origins, are able to differentiate into diverse types of cells including adipocytes, chondrocytes, osteoblasts, and myocytes [28]. In addition, MSCs have immunomodulatory properties to regulate various cells involved in immune responses, such as dendritic cells (DCs), lymphocytes, macrophages, mast cells, neutrophils, and natural killer (NK) cells [24]. On these bases, MSCs have been spotlighted as potent cell therapeutics for various diseases over the last decades.

In the reported preclinical studies of MSC-exosomes, MSCs were isolated from various tissues/cells in the following order: BM (51%), umbilical/placental tissues (23%), adipose tissue (13%), derived from ESCs or iPSCs (8%), and others (5%) [29]. Since characteristics and functionality of MSCs depend on their origins, it is obvious that those of MSC-exosomes vary according to the origin of MSCs. However, comparative studies of MSC-exosomes by their tissue origin are still limited, and only a few reports have compared different MSC-exosomes within the same study (Table 1) [30,31,32,33,34,35]: (1) human adipose tissue-derived MSC (ASC)-exosomes exhibited a higher activity of neprilysin, an amyloid β (Aβ) peptide degrading enzyme in the brain, than human bone marrow MSC (BM-MSC)-exosomes, suggesting the therapeutic relevance of ASC-exosomes in Alzheimer’s disease [30]; (2) human BM-MSC-EVs and Wharton’s jelly MSC (WJ-MSC)-EVs decreased cell proliferation and induced apoptosis, while ASC-EVs increased cell proliferation and had no apoptotic effect in U87MG glioblastoma cells [31]. However, the effects of MSC-exosomes on cancer cells are controversial [36]. For example, ASC-exosomes have been reported to have anti-cancer activity on prostate cancer both in vitro and in vivo [37]; (3) human menstrual fluid MSC (MenSC)-exosomes and BM-MSC-exosomes promoted neurite growth both in cortical and sensory neurons, while human chorion MSC-exosomes and UC-MSC-exosomes did not. This suggests that appropriate selection of MSC sources might be essential for the treatment of neurodegenerative diseases [32]; (4) human iPSC MSC (iMSC)-exosomes and synovial membrane MSC (SM-MSC)-exosomes both attenuated osteoarthritis (OA) in a murine model, but iMSC-exosomes had a superior therapeutic effect compared to SM-MSC-exosomes [33]; (5) a study comparing canine MSCs reported that BM-MSCs released a higher level of secretome, including exosomes, than ASCs did [34]; and (6) human amniotic fluid MSCs (AF-MSCs) released a higher amount of exosomes than BM-MSCs [35]. However, it is difficult to directly compare the results between the above studies, since they were not performed with comparable processes or methods for isolation, characterization, and efficacy evaluation for exosomes. In addition, variations from different donors or preparation methods for MSCs remain a prominent challenge [38,39]. Nevertheless, it is suggested that MSC-exosomes might exhibit different properties and efficacies depending on the origin of MSCs. Therefore, biological differences such as the origin of MSCs and efficacy of their exosomes should be considered for specific clinical applications.

## 3. Quality Control of EVs for Development of Therapeutic EVs

It is of importance to manufacture clinical-grade EVs with a good manufacturing practice (GMP)-compliant process and quality control (QC) for the development of EV-based therapeutics [40,41,42]. Appropriate QC is also crucial for reproducible studies in academic settings. Recently, the International Society for Extracellular Vesicles (ISEV) proposed a series of the Minimal Information for Studies of Extracellular Vesicles (MISEV), finalized as MISEV2018 [43,44,45]. The Korea Ministry of Food and Drug Safety (MFDS) published the world’s first guideline for EV therapy products, entitled the Guideline on Quality, Non-clinical, and Clinical Assessment of Extracellular Vesicles Therapy Products [46]. As shown in Table 2, most of the criteria in these guidelines are similar [1] and have been already been applied in GMP settings [42,47,48]. Routine QC criteria include the determination of the quantity, size, identity, and purity of EVs.

### 3.1. EV Quantity and Size

Both the MISEV2018 and the MFDS guidelines recommend using at least two different methods for determining the quantity of EVs [45,46]. Quantification of EVs can be achieved by measuring the total amounts of proteins, lipids, or RNAs, since EVs consist of all these molecules. These methods, however, do not provide the information on the number of EV particles. Several methods are available to measure the number and size of particles, including nanoparticle tracking analysis (NTA), resistive pulse sensing (RPS), and dynamic light scattering (DLS). The most widely used method is NTA [42,47,48,49,50,51,52,53]. NTA determines the number and size of particles by tracking the Brownian motion of single particles in an aqueous solution [54]. However, NTA suffers from a low resolution of poly-dispersed samples and high variations, such as inter-device, inter-assay, and intra- and inter-individual variations [55,56,57]. In addition, NTA does not differentiate EVs from other nanoparticles such as protein aggregates. Recently, instruments for fluorescence NTA have been introduced to detect fluorescently labeled EVs with specific antibodies [58]. Quantification of EVs, however, remains extremely challenging. New technologies and instruments have been introduced annually, especially during the ISEV conference, such as nano flow cytometry [59,60], direct stochastic optical reconstruction microscopy [61], ExoCounter with the optical disc technology [62], and imaging flow cytometry [63]. Although it will take some time to develop fully GMP-compatible instruments, the great strides forward in methodologies for the quantification of EVs are expected to result in the overcoming of current hurdles in the near future.

### 3.2. EV Identity

A variety of proteins have been reported to be associated with EV, especially exosomes, including tetraspanins (CD9, CD63, and CD81), Annexins, Flotillin, ALG-2-interacting protein X (Alix), and tumor susceptibility gene 101 (TSG101) protein [45,64]. Proteins such as CD9, CD63, CD81, TSG101, and Alix are recommended as specific markers for exosomes since they are known to be highly enriched in exosomes compared to the originating cells [45,64,65,66]. In addition, because Alix and TSG101 are involved in the formation of multivesicular bodies (MVBs), presence of these proteins is essential to support the endocytic origin of exosomes [43,45,64]. For QC, at least semi-quantitative methods are recommended to detect these proteins in exosomes [46]. The enzyme-linked immunosorbent assay (ELISA) and flow cytometric analysis are each suitable for both GMP-compliant facilities and general academic labs. Although Western blotting has been widely used in the academic labs, this method is limited by lack of appropriate quantification and method validation [67].

### 3.3. EV Purity

Purity of EVs is also a critical criterion for QC. A simple method to monitor purity of EVs is to determine the particle-to-protein, protein-to-lipid, or RNA-to-particle ratios [45]. The absence of intracellular proteins, such as histones, lamin A/C, GRP94 (i.e., HSP90B1), GM130 (i.e., GOLGA2), and cytochrome C (i.e., CYC1), is another important criterion to determine the purity of EVs or exosomes, since these proteins are not enriched in exosomes due to their strict cellular localization [43,45]. Impurities from cell culture process including antibiotics and serum should also be analyzed to monitor the removal of potential hazardous substances [46]. Every batch of EVs should be qualified by routine QC before being used for therapeutic purposes or functional assays, even in the academic labs, to ensure reproducibility.

### 3.4. Potency Assays

Potency assays are the most important QC criterion to predict efficacy of EVs in vivo. Regulatory authorities such as the US Food and Drug Administration (FDA) recommend using appropriate potency tests for cellular and gene therapy products [68]. The MISEV2018 and the MFDS guidelines also recommend including potency assays for EV QC [45,46]. Potency is defined as “the specific ability or capacity of the product, as indicated by appropriate laboratory tests or by adequately controlled clinical data obtained through the administration of the product in the manner intended, to effect a given result” [68]. Many biological and biochemical assays have been reported to demonstrate the potency of EVs or exosomes [69,70]. Since quantification of EVs remains challenging, establishment of an appropriate potency assay would be an invaluable tool to monitor batch-to-batch consistency and determine the dose of EVs [71]. Although ideal potency assays should represent the MoA, it is difficult to set-up an appropriate potency assay with single biochemical or isolated cell-based assays due to the difficulty in the identification of single bioactive substances in the complex cargo of EVs. As an example, it is hard to mimic the complex immune responses in vivo with in vitro cell-based assays [70,71,72,73].

## 4. Anti-Inflammation and Immunomodulation by MSC-Exosomes

Immune cells secrete soluble factors such as inflammatory cytokines and mediators, which can contribute in the event of inflammation [74,75]. In particular, pro-inflammatory cytokines, including tumor necrosis factor (TNF)-α, interleukin (IL)-6, and IL-1β, are mainly produced by activated macrophages. These cytokines play important roles in the upregulation of inflammatory responses such as activation of macrophages and recruitment of additional immune cells [74,75]. In contrast, anti-inflammatory cytokines are produced by regulatory T cells (Tregs), helper T (Th)2 cells, alternatively activated macrophages, and monocytes, which control the inflammatory responses and immunity [75,76]. Major anti-inflammatory cytokines include IL-1 receptor agonist (IL-1RA), IL-4, IL-10, and transforming growth factor (TGF)-β [76]. These cytokines inhibit the Th1 responses and production of pro-inflammatory cytokines [76].

Inflammation is a mechanism of innate immunity in response to harmful stimuli, including pathogens, damaged cells, or irritants, and typically manifests as heat, pain, redness, swelling, and loss of function [77]. Uncontrolled chronic inflammatory responses are associated with diverse inflammatory diseases such as allergy, asthma, autoimmune diseases, inflammatory bowel disease (IBD), OA, atherosclerosis, and hepatitis [77,78,79]. In addition, many scientists now consider inflammation as the root cause of most chronic diseases such as heart attacks, strokes, type 2 diabetes, Alzheimer’s disease, and even cancer [80,81]. Therefore, regulation of inflammation is an important therapeutic target to treat inflammatory diseases. It has been demonstrated that MSCs have property of intrinsic immunosuppressive capabilities to alleviate inflammation and immune responses [82]. MSC-exosomes can be an excellent alternative to MSC cell therapy since MSC-exosomes possess similar biological functions to the originating cells, while they are more stable and have lower immunogenicity compared to their originating cells [83]. In fact, anti-inflammatory and immunomodulatory functions of MSC-exosomes have been extensively reported (Table 3) [21,84,85,86,87,88,89,90,91,92,93,94,95,96,97,98,99,100,101,102,103,104,105,106,107,108,109,110,111,112,113,114,115,116,117,118,119,120,121,122,123,124,125,126,127,128,129,130,131,132,133,134,135,136,137,138,139,140,141,142,143,144,145,146,147,148,149,150,151].

### 4.1. Macrophage Polarization

There is accumulating evidence that MSC-exosomes promote macrophage polarization from M1 toward M2. M1 macrophages are characterized by the expression of a broad spectrum of pro-inflammatory cytokines and chemokines, such as IL-1β, IL-12, and TNF-α. By contrast, the M2 macrophage phenotype is induced by Th2 cytokines and leads to secretion of anti-inflammatory factors, such as IL-10 and TGF-β, and M2 markers such as IL-1RA, CD163, and C-C motif chemokine 22 (CCL22) [152]. It has been reported that human BM-MSC-exosomes and jaw bone marrow MSC (JM-MSC)-exosomes promote cutaneous wound healing [86], and ameliorate bronchopulmonary dysplasia (BPD) [86] through macrophage M2 polarization. The miR-223 contained in exosomes alleviated inflammation and accelerated wound healing by inducing macrophage M2 polarization. Co-culture with BM-MSC-exosomes increased the expression of miR-223 and decreased the expression of PBX/knotted homeobox 1 (PKNOX1) protein, an important regulator of macrophage polarization, in macrophages isolated from peripheral blood mononucleated cells (PBMCs). Besides, after co-culture with BM-MSC-exosomes CD206-positive macrophages were elevated, and miR-223 inhibitors reversed this elevation [85]. In a high-fat diet (HFD) mouse model, miR-223 deficiency enhanced infiltration of M1 macrophage, and increased production of pro-inflammatory cytokines, but decreased M2-associated biomarkers including peroxisome proliferator-activated receptor γ (PPARγ) and arginase 1 (ARG1) [153]. Another study elucidated that human UC-MSC-exosomes also promote M2 macrophage activation and regulate diabetic cutaneous wound healing [87]. Compared to those from unconditioned UC-MSCs, exosomes from LPS-preconditioned UC-MSCs contained a high level of let-7b, ameliorated inflammation, and promoted wound healing more intensely. UC-MSC-exosomes decreased toll-like receptor 4 (TLR4) and phospho (p)-p65 proteins regardless of LPS preconditioning. After treatment of LPS-preconditioned UC-MSC-exosomes, ARG1, an M2 macrophage marker, was increased, and inducible nitric oxide synthase (iNOS), an M1 macrophage marker, was decreased [88]. The let-7b targets TLR4, activation of which leads to activation of nuclear factor κB (NF-κB). Additionally, let-7b downregulates the expression of cyclooxygenase-2 (COX-2) and cyclin D1 proteins [154]. It was revealed that UC-MSC-exosomes suppress inflammation and promote wound healing by inducing secretion of cytokines from M2 macrophages in rats with severe burn-induced skin inflammation through downregulation of TLR4, NF-κB, and p-p65 expression [89]. A higher level of miR-181c was observed in UC-MSC-exosomes compared to in human dermal fibroblast (HDF)-exosomes. The expression level of miR-181c was decreased by burn injury and was increased after treatment of UC-MSC-exosomes in the cutaneous wound. In addition, treatment of UC-MSC-exosomes reduced the expression of TNF-α and IL-1β and increased the expression of IL-10. These effects were strengthened by exosomes derived from miR-181c-overexpressed UC-MSCs [88]. In an experiment conducted in mouse astrocytes, the expression level of miR-181c was decreased by LPS, a TLR4 receptor ligand. Overexpression of miR-181c increased IL-10 secretion induced by LPS [155]. In primary microglia, oxygen-glucose deprivation (OGD) upregulated TLR4, while miR-181c reversed this upregulation. The miR-181c also downregulated NF-κB and pro-inflammatory cytokines such as TNF-α, IL-1β, and iNOS induced by OGD [156]. In addition, it was found that human MenSC-exosomes induced macrophage M2 polarization, which was confirmed by the increased ARG1/iNOS ratio, which led to the alleviation of inflammation in the diabetic cutaneous wound [89].

Moreover, exosomes derived from various MSCs also play an important role in promoting activation of M2 macrophages in other inflammatory diseases as well as cutaneous wounds. It was found that mouse BM-MSC-exosomes relieve inflammation in atherosclerosis via macrophage M2 polarization in vivo through the let-7/high mobility group AT-Hook 2 (HMGA2)/NF-κB pathway [90]. An enrichment of the let-7 family was found in BM-MSC-exosomes, and treatment of BM-MSC-exosomes upregulated the let-7 level in ApoE–/– mice [90]. Zhao et al. revealed that mouse BM-MSC-exosomes also attenuated myocardial ischemia-reperfusion (IR) injury through polarizing macrophages toward M2 phenotypes (iNOS-CD206+), and increasing IL-10 and ARG1, which are regulated by miR-182 targeting TLR4 [91]. Human BM-MSC-exosomes have been reported to reduce dextran sodium sulfate (DSS)-induced IBD in mice through the polarization of M2b macrophages in a metallothionein-2 (MT2A)-dependent manner [92]. Another report revealed that mouse ESC-exosomes improved cardiomyopathy by increasing M2 macrophages and IL-10 release [157]. Additionally, it was reported that rat ASC-exosomes ameliorated myocardial infarction by promoting M2 macrophage polarization, which is regulated by increasing sphingosine-1-phosphate receptor 1 (S1PR1) [93]. The importance of the sphingosine 1-phosphate (S1P)/sphingosine kinase 1 (SphK1)/S1PR axis was further confirmed by silencing of S1PR1, which abolished the decrease of hypoxia-induced apoptosis by ASC-exosomes in H9c2 cells. Similarly, human ASC-exosomes induced M2 macrophage markers in human PBMCs [94]. Heo et al. revealed that human ASC-exosomes also induce M2 macrophage phenotype by confirming the increased level of transcription factors (e.g., signal transducer and activator of transcription 6 (STAT6), MAF BZIP transcription factor B (MafB), etc.), which led to regulating immunomodulatory and anti-inflammatory effects such as increased Tregs and anti-inflammatory cytokines (e.g., IL-10 and TNF-α-stimulated gene-6 (TSG-6)) [94]. Mouse ASC-exosomes also induced M2 macrophage polarization and reduced inflammation of white adipose tissues (WAT) in obese mice [96]. These effects are dependent on a transcription factor, STAT3, in ASC-exosomes. Furthermore, ASC-exosome-educated M2 macrophages induced proliferation of ASCs themselves and production of lactate from ASCs, which further promoted WAT beiging [95]. However, further studies are needed to understand the detailed underlying molecular mechanism for the regulation of M2 macrophage polarization by MSC-exosomes.

### 4.2. T Cell Regulation

MSC-exosomes also modulate functions or activities of T cells (Table 3). BM-MSC-exosomes were reported to convert Th1 to Th2, and reduce Th17 differentiation in PBMCs [97]. More importantly, BM-MSC-exosomes increased the level of Tregs in PBMCs. These effects might be mediated by suppression of pro-inflammatory cytokines such as TNF-α and IL-1β, and an increase of anti-inflammatory cytokine TGF-β [9]. Another report also revealed that BM-MSC-exosomes modulate immune reactions in PBMCs from asthmatic patients [98]. The proliferation and immune-suppression capacity of Tregs was promoted by BM-MSC-exosomes through upregulation of IL-10 and TGF-β1 in PBMCs. Tregs was also induced by exosomes derived from TGF-β/IFN-γ-stimulated UC-MSCs [99]. The proposed mechanism of this Treg regulation is an antigen presenting cell (APC)- but not CD4+ T cell-dependent pathway [97]. A previous report demonstrated that differentiation of Tregs is mediated by activated APCs, which is induced by ESC-MSC-exosomes in a myeloid differentiation primary response 88 (MYD88)-dependent manner [100]. It has been also reported that mouse ASC-exosomes induce the increase of Tregs population in the splenic mononuclear cells from mice with streptozotocin-induced autoimmune type 1 diabetes mellitus [100]. Upregulation of Tregs has been also reported in a multiple sclerosis (MS) mouse experimental autoimmune encephalomyelitis model by human BM-MSC-exosomes [101], and a concanavalin A (Con A)-induced mouse liver injury model by mouse BM-MSC-exosomes [102]. Downregulation of proliferation of activated T and B lymphocytes by BM-MSC-exosomes has been also reported [103]. Of note, studies by Del Fattore et al. and Di Trapani et al. have shown that EVs from BM-MSC suppress T cell proliferation indirectly by induction of Treg differentiation, unlike MSCs, which directly suppress T cell proliferation [103,104]. In addition, UC-MSC-EVs purified by size exclusion chromatography only showed an inhibitory effect on T cell proliferation and did not induce cytokine response and monocyte polarization [105]. Further studies are needed to elucidate the molecular mechanism of these regulations by MSC-exosomes.

### 4.3. Inflammation in Skin

It was reported that human BM-MSC-exosomes reduce photoaging and inflammation in mice, which might be helpful to prevent and treat cutaneous aging [107]. Human ASC-exosomes were reported to enhance neovascularization and the survival of the skin flap in a rat IR injury of the flap transplantation model by reducing inflammation and apoptosis [108]. In this experimental setting, ASC-exosomes derived from H_2_O_2_-preconditioned ASCs had better outcomes compared to those from unconditioned ASCs. Regulation of inflammation is also important to treat atopic dermatitis (AD), a representative skin inflammatory disease. It has been demonstrated that human ASC-exosomes can ameliorate AD in two distinct mouse models via reducing pathological symptoms and expression of multiple cytokines such as IL-4, IL-5, IL-13, IL-17, IL-23, IL-31, TNF-α, IFN-γ, and thymic stromal lymphopoietin (TSLP) [20,109]. Th2 cytokines, such as IL-4, IL-5, IL-13, and IL-31, mainly produced by activated Th2 cells, are crucial contributing factors in the development of allergic inflammation in the skin [158,159]. Notably, Th2 cytokines including IL-4, IL-13, and IL-31 are therapeutic targets for AD [160]. Additionally, ASC-exosomes also reduced the infiltration of inflammatory dendritic epidermal cells (IDECs, CD86+, and CD206+), which led to release of pro-inflammatory cytokines in lesional skin of AD [20]. Taken together, MSC-exosomes are key players in skin regeneration by promoting macrophage M2 polarization with anti-inflammatory properties and reducing pro-inflammatory cytokine-releasing cells such as M1 macrophages and IDECs.

### 4.4. Immunomodulation in Other Inflammatory Diseases

Immunomodulation by MSC-exosomes was also reported in various inflammatory disease models. Examples are as follows: (1) Exosomes from melatonin-preconditioned rat BM-MSCs reduced the kidney injury in a rat renal IR injury model by decreasing oxidative stress and apoptosis, increasing anti-oxidant and anti-apoptotic proteins, and enhancing angiogenesis [110]. In addition, mouse BM-MSC-exosomes reduced the renal IR injury in a CCR2-dependent manner [111]. Human UC-MSC-exosomes have been also reported to reduce cisplatin-induced acute kidney injury (AKI) in rats in an autophagy-dependent manner [112]; (2) Human UC-MSC-exosomes reduced the experimental autoimmune uveitis in rats [113]; (3) Human placenta-derived MSC (PL-MSC)-exosomes reduced the tissue fibrosis and inflammation in a mouse Duchenne muscular dystrophy (DMD) model partly through the delivery of miR-29c [114]; (4) Human UC-MSC-exosomes improved the pathology of lung, cardiac, and brain in neonatal mice with BPD by reducing the pulmonary inflammation and alveolar-capillary leak potentially through the delivery of TSG-6 [115] or macrophage M2 polarization [86]; (5) Targeted delivery of mouse BM-MSC-exosomes by rabies viral glycoprotein (RVG) peptide improved the cognitive function of transgenic APP/PS1 mice by reducing plaque deposition, the level of Aβ, activation of astrocytes, and the expression of pro-inflammatory cytokines TNF-α, IL-β, and IL-6, while increasing the levels of IL-10, IL-4, and IL-13 [116]; (6) Human BM-MSC-EVs improved the neurological impairment and long-term neuroprotection in stoke mice by attenuating the post-ischemic immunosuppression and lymphopenia, and as well as stimulating neurogenesis and angiogenesis [117]; and (7) Mouse BM-MSC-exosomes decreased the threshold for thermal and mechanical stimuli in a mouse diabetic peripheral neuropathy model by regulating multiple factors involved in macrophage polarization through the delivery of miRNAs targeting the TLR4/NF-κB signaling pathway [118]. Other inflammatory diseases, which can be modulated by MSC-exosomes or MSC-EVs, include OA [119,120], intervertebral disc degeneration (IVDD) [123], spinal cord injury [124,125,126], myocardial infarction [127,128], acute lung injury (ALI) [129,130,131], idiopathic pulmonary fibrosis (IPF) [132], hepatic IR injury [133], liver fibrosis [134], acute liver failure [135], IBD [92,136], necrotizing enterocolitis [137], abdominal aortic aneurysm [139], brain injuries [139,140,141,142,143], urethral stricture [144], status epilepticus (SE) [145,146], retinal injuries [147,148], sepsis [150], and graft-versus-host disease (GvHD) [150]. The immunomodulation of MSC-exosomes was highlighted in their first clinical application in an allogeneic setting to a patient suffering from steroid refractory GvHD [151]. In this study, MSC-exosomes modulated the status of the patient’s immune cells. The differentiation of Tregs by MSC-exosome-mediated APC activation might contribute to suppression of GvHD [99].

In summary, MSC-exosomes or MSC-EVs suppress inflammatory responses in diverse disease settings by inducing polarization and differentiation of M2 macrophages and Tregs. Although exact cargo compositions and MoA of exosomes need to be further studied, mounting evidence suggests that MSC-exosomes have similar anti-inflammatory and immunomodulatory properties of MSCs, which could be beneficial for the treatment of inflammatory and autoimmune diseases, as well as for skin regeneration. However, MSC-exosomes may also possess distinct immunomodulatory mechanisms from those of MSCs, which needs to be further elucidated to facilitate application in clinical settings.

## 5. Anti-Aging Effects of MSC-Exosomes

Aging, defined as irreversible deterioration of physiological processes of organisms over time, is characterized by nine hallmarks: cellular senescence, mitochondrial dysfunction, deregulated nutrient sensing, epigenetic alterations, telomere attrition, genomic instability, altered intercellular communication, and stem cell exhaustion [161,162]. Among these, cellular senescence has recently been focused on as one of the key factors in the complex aging process as it is interlinked with other hallmarks [163]. Senescent cells are accumulated in tissues of vertebrates with age. Interestingly, removal of senescent cells in animals results in the delayed onset of age-associated diseases [164,165,166,167,168]. Senescence is characterized by a stable cell-cycle arrest in the G1 phase and an inflammatory response called senescence-associated secretory phenotype (SASP), which modifies the microenvironment around senescent cells [161]. Senescence is induced by intracellular and extracellular stresses, including replicative stress, DNA damage, oncogene activation, telomere damage or shortening, inflammation, mitochondrial dysfunction, oxidative stress, and drug insults, to eliminate damaged cells, and prevents potential malignant cell transformation [161,169]. Components of the SASP include growth factors, pro-inflammatory cytokines, chemokines, and extracellular matrix remodeling enzymes [170,171,172]. SASP contributes to inflammaging, a term coined by Franceschi et al. in 2000, which describes low-grade, controlled, asymptomatic, chronic, and systemic inflammation associated with aging processes [173]. Indeed, many evidences point out that inflammaging may ultimately lead to age-related diseases [174,175,176]. Thus, interventions that suppress SASP and inflammaging processes may hold potential to alleviate various chronic diseases [177]. In addition, senescent cells display the expression of senescence-associated β-galactosidase (SA-β-gal), increases of mRNAs/proteins including p53, p21, p16, and γ-H2AX, and a decrease in cell proliferation [161].

### 5.1. EVs in Senescence

EVs or exosomes have a role in both transferring the senescence phenotype and alleviating or even rejuvenating senescence cells, depending on their originating cells. Studies suggest that EVs or exosomes act as new components of the SASP and age-related disease markers [169,170,171]. Age-related changes of EVs or exosomes have been reported to result in the following: (1) an increase in the number of EVs or exosomes released during senescence of fibroblast, epithelial cells, and cancer cells [178,179]; (2) a decrease in the levels of circulating EVs with age, at least from the 30s to 60s in humans, as well as in mice and rats [180,181,182]; and (3) changes of EV or exosome composition (miRNAs, proteins, or lipids) associated with aging or senescence [171,183,184,185,186,187,188,189]. In fact, EVs or exosomes mediate paracrine senescence, transmitting senescence from senescent or diseased cells to normal cells, in both normal and disease conditions [169,190,191,192,193,194,195]. This paracrine senescence is positively correlated with the uptake of exosomes by target cells and is prevented by inhibition of exosome generation [169].

It has also been reported that various long noncoding RNAs (lncRNAs) are enriched in exosomes from senescent cells and accumulating evidence shows that these RNAs may contribute to the progression of age-related diseases such as atherosclerosis, type 2 diabetes, osteoporosis, OA, rheumatoid arthritis, Parkinson’s disease, and multiple sclerosis [196]. It has also been reported that various long noncoding RNAs (lncRNAs) are enriched in exosomes from senescent cells, and accumulating evidence shows that these RNAs may contribute to the progression of age-related diseases such as atherosclerosis, type 2 diabetes, osteoporosis, OA, rheumatoid arthritis, Parkinson’s disease, and multiple sclerosis. For instance, in atherosclerosis, monocytes exposed to oxidized low-density lipoprotein (oxLDL) drives progression of the disease. A study by Chen et al. has shown that THP-1, a monocyte cell line, treated with oxLDL shows significant upregulation of exosomal lncRNA GAS5, and these exosomes cause apoptosis of endothelial cells [197]. The role of exosomal lncRNA was also highlighted by Ruan et al. In this study, it was found that exosomal lncRNA-p3134 contents in diabetic patients were higher than those in non-diabetic subjects [198]. Senescent cells also exert effects by transferring protein cargo. For instance, exosomes from drug-induced senescent multiple myeloma cells promote activation and proliferation of NK cells by transferring IL-15RA and IL-15 [199]. Taken together, EVs from senescent cells may serve as disease markers.

### 5.2. Anti-Aging Effects

It has been elusive that circulating mediators are responsible for rejuvenating multiple tissues of old organisms by parabiosis of young organisms [200]. Very recently, it was demonstrated that EVs from young mice plasma extend the lifespan of old mice by delaying aging through exosomal nicotinamide phosphoribosyl transferase (eNAMPT) [201]. Another study also reported that exosomes from young mice could transfer miR-126b-5p to tissue of old mice, and reverse the expression of aging-associated molecules such as p16, mTOR, IGF-1R, and telomerase-related genes including *Men1*, *Mre11a*, *Tep1*, *Terf2*, *Tert*, and *Tnks,* in aged mice [202]. Another report revealed that EVs derived from serum of young mice attenuated inflammaging in old mice by partially rejuvenating aged T-cell immunotolerance [203]. Implantation of hypothalamic stem/progenitor cells, which were genetically engineered to survive from aging-related hypothalamic inflammation, was reported to induce retardation of aging and extension of lifespan in mid-aged mice [204].

More importantly, growing evidence suggests that cellular senescence can be alleviated or reversed by EVs or exosomes derived from stem cells (Table 4) [205,206,207,208,209,210,211,212,213,214]. Human ASC-exosomes reduced the high glucose-induced premature senescence of endothelial progenitor cells (EPCs) and enhanced wound healing in diabetic rats [205]. In the same study, overexpression of nuclear factor erythroid 2-related factor 2 (NRF2) in human ASC-exosomes further reduced premature senescence of EPCs, and promoted wound healing in diabetic rats by modulating the expression of various proteins [205]. Since high glucose in diabetic patients induces reactive oxygen species (ROS) and inflammation, which promotes senescence and impairs function of EPCs, reduced senescence of EPCs by ASC-exosomes may be beneficial for the treatment of diabetic foot ulcers [205]. It has also been reported that human ASC-exosomes contain lnRNA MALAT1 and recover function of motor behavior with reduction of cortical brain injury in a rat traumatic brain injury model [142]. Regarding this, a study revealed that the MALAT1 expression is reduced in aged mice and that treatment of human UC-MSC-exosomes containing MALAT1 prevents aging in D-galactose (gal)-treated mice and senescence in H_2_O_2_-treated H9C2 cardiomyocytes [206]. MALAT1 is one of the candidates for anti-aging effects in stem cell-derived exosomes, since MALAT1-knockdown in UC-MSCs abolished these effects of UM-MSC-exosomes. Similarly, exosomal miR-146a was known to negatively regulate senescence of MSCs by targeting the NF-*κ*B signaling [191]. Recently, miR-146a in AF-MSC-exosomes was reported to reduce LPS-induced inflammation in the human trophoblast cells [215]. The miR-146a is also known to be enriched in human UC-MSC-exosomes by TNF-α-pre-conditioning, and mediate anti-inflammatory effects in a rat urethral stricture model [145]. Antioxidant enzymes peroxiredoxins (PRDXs) were reported as being highly enriched in iPSC-EVs and BM-MSC-EVs [208]. Transferring of PRDXs by these EVs resulted in alleviation of cellular aging phenotypes such as increases of SA-β-gal, p21, p53, IL-1α, IL-6, and γ-H2AX in both replicative and genetically induced senescent MSCs [209]. Interestingly, proteomic analysis revealed that ASC-exosomes also contain PRDXs such as PRDX1, PRDX4, and PRDX6 [109]. Human ASC-exosomes were also reported to reduce IL-1β-induced senescence in osteoblasts from OA patients [209]. In this study, ASC-exosomes reduced not only the levels of SA-β-gal, γ-H2AX, and IL-6 protein, but also the levels of prostaglandin E2, oxidative stress, and mitochondrial membrane potential. It has been reported that miR-214 in exosomes prevents senescence of endothelial cells by repressing the expression of ataxia telangiectasia mutated (ATM) protein by targeting the 3’-untranslated region (UTR) of its mRNA [216]. Interestingly, the next generation sequencing (NGS) analysis revealed that ASC-exosomes also contain miR-214 (Ha et al. unpublished observation).

Mouse miR-291a-3p was identified to target TGF-β2 receptor and as a cargo of mouse ESC-exosomes [211]. Treatment of mouse ESC-exosomes reduced the SA-β-gal expression and promoted cell proliferation and migration of replicative or adriamycin-induced senescent HDFs [211]. It was reported that human ESC-exosomes inhibited D-gal-induced senescence of human vascular endothelial cells (HUVECs) [212]. Treatment of ESC-exosomes resulted in a decrease in SA-β-gal activity, p16 and p21 protein levels, and ROS in HUVECs, and an increase in cell proliferation, migration, and tube formation of HUVECs. The miR-200a in ESC-exosomes reduced the level of Kelch-like ECH-associated protein 1 (KEAP1) by targeting the 3’-UTR of *KEAP1* mRNA. As a result, the level of NRF2, a master regulator of anti-oxidative responses [217], was increased to induce the expression of its downstream targets such as heme oxygenase 1 (HO1), superoxide dismutase (SOD), and catalase (CAT) [213]. ESC-exosomes promoted pressure ulcer healing in D-gal-induced aged mice by reducing endothelial senescence and increasing angiogenesis [212]. Human iPSC-exosomes were reported to protect HDFs from UVB damage, reduce the senescence-associated MMP-1/3 expression, and induce synthesis of collagen type I in both UVB-damaged and senescent HDFs [214]. Human iPSC-exosomes were also reported to reduce SA-β-gal and increase cell viability and tube formation of high glucose-injured HUVECs with unknown mechanism [214]. Exosomes from various cells are also useful as a delivery vehicle of biomolecules to suppress senescence. The miR-675 was discovered as a candidate marker for aging [207]. Delivery of miR-675 through UC-MSC-exosomes reduced the SA-β-gal expression, and the levels of p21 and TGF-β1 proteins in H_2_O_2_-induced senescent H9C2 cells by targeted downregulation of TGF-β1. Additionally, miR-675-UC-MCS- exosomes promoted perfusion in ischemic hindlimb by inhibiting the expression of both mRNAs and proteins of p21 and TGF-β1 [207]. Another study reported that exosomes derived from Wnt4-overexpressed mouse thymic epithelial cells (TECs) inhibited dexamethasone-induced aging phenotypes in TECs [218].

Taken together, MSC-exosomes confer anti-senescence effects through their unique miRNA, lnRNA, and enzyme contents. By inducing proliferation and reducing SASP in senescent cells, they hold great potential to reduce senescent cells in tissues. Since removal of senescent cells from tissues was reported to create a pro-regenerative environment [168] and tissue homeostasis [166], application of MSC-exosomes to remove the senescent cells may be a preferable approach to induce the regeneration or rejuvenation of tissues.

## 6. Cutaneous Wound Healing by MSC-Exosomes

A wound is a type of injury in skin. An open wound is caused by a tear, cut, or puncture, and a closed wound is caused by blunt trauma [219]. Cutaneous wounds can be classified into acute and chronic wounds [220]. Acute wounds are highly prevalent from a loss of dermis and epidermis caused by mechanical, chemical, biological, or thermal injuries. Chronic wounds, on the other hand, are common comorbidities of complex diseases such as obesity, diabetes, and vascular disorders. Four categories of chronic wounds include pressure ulcers, diabetic ulcers, venous ulcers, and arterial insufficiency ulcers according to the Wound Healing Society [221]. Since chronic wounds do not heal within three months, they are considered as non-healing wounds [222,223]. Another major medical issue is pathological wound healing and scar formation, which cause both physiological and psychological challenges [224]. The annual Medicare cost for the treatment of acute and chronic wounds was estimated at from $28.1 to $96.8 billion [225]. In addition, the annual product market for wound care is estimated to reach $15 to $22 billion by 2024 [225].

Cutaneous wound healing is the complex process of restoring the injured skin. It consists of four phases: the homeostasis, inflammatory, proliferative, and remodeling phases [226,227,228]. Responses in these phases are tightly coordinated to secure vital skin barrier functions [224]. However, the mechanism of cutaneous wound healing and the interplays between a variety of cells during the wound healing process have been only partly delineated [229]. Many cell types interact with each other in a highly sophisticated sequence during the cutaneous wound healing process as follows [230]: (1) the platelets initiate the formation of the blood clots, which consist of platelets, red blood cells, and extracellular matrix molecules in the first homeostasis phase; (2) neutrophils, monocytes, as well as macrophages are major players during the inflammatory phase. Chemotactic factors released by neutrophils attract monocytes and cytokines from macrophages and stimulate migration of fibroblasts to enter the injured site from the surrounding normal tissues; (3) angiogenesis and vascularization of endothelial cells provide oxygen supply to support proliferation of migrated cells in the wound site during the proliferative phase. Fibroblasts also differentiate into myofibroblasts to generate a tensile strength in the wound. In addition, fibroblasts secrete growth factors, which activate migration and proliferation of keratinocytes. Reepithelialization is completed by stopping migration of cells by contact inhibition [230]; and (4) remodeling through apoptosis of fibroblasts, myofibroblasts, and other cells, and degradation of extracellular matrix occur during the wound scar remodeling phase, which spans months to years. Adverse scarring, caused by aberrant wound healing, includes chronic non-healing wounds and pathological scarring such as hypertrophic scars and keloids, and it affects millions of people globally since currently no effective treatment option is available [224]. The prevention or reduction of scars is also an important issue to solve in the regenerative aesthetics [231].

MSC-EVs or MSC-exosomes orchestrate all phases of skin wound healing because of their ability to modulate inflammation, activate migration and proliferation of various cells including immune cells, fibroblasts, and keratinocytes, and even ameliorate scarring (Table 5) [85,87,88,205,226,231,232,233,234,235,236,237,238,239,240,241,242,243,244,245]. As an example, complete reepithelialization was reported in a rabbit cutaneous wound healing model by EVs from rabbit ASCs and BM-MSCs with an unknown mechanism [232]. Human ASC-EVs were also reported to enhance cutaneous wound healing in rat [233].

### 6.1. Homeostasis Phase

During the homeostasis phase, the formation of blood clots by platelets protects the injured site. Up to now, no direct evidence has been available that shows the involvement of MSC-exosomes in blood clotting during wound healing. A recent result might suggest the potential benefit of MSC-exosomes on blood clotting in the wound healing process; human UC-MSC-EVs have been reported to induce blood coagulation in vitro [244]. However, further studies are required to analyze the effects of MSC-EVs or MSC-exosomes in blood clotting in both healthy and disease conditions.

### 6.2. Inflammatory Phase

Regulation of inflammation is also important in skin regeneration during the wound healing process. Although inflammation is one phase of the normal skin repair cascade, the prolonged inflammation is harmful and may cause excessive scarring [245]. The prolonged inflammation happens mainly in chronic or burn wounds [226,246] and it is of importance to appropriately transit from inflammatory to proliferative phases in normal wound healing [247]. Macrophages are crucial in the wound healing process, which should appropriately transition from M1 to M2 macrophages [248,249]. M2 macrophages have anti-inflammatory properties, which are promoted in order to repair wounds in the latter phases of skin wound healing [248,249]. As mentioned earlier, MSC-exosomes promote the polarization of macrophages from M1 to M2 in cutaneous wound healing models (see 4. Anti-inflammation and immunomodulation by MSC-exosomes): (1) human BM-MSC-exosomes and JM-MSC-exosomes promote cutaneous wound healing in mice by transferring miR-223 [85]; (2) human UC-MSC-exosomes promoted diabetic cutaneous wound healing in rats by delivering let-7b [88]; and (3) human UC-MSC-exosomes enhanced the wound healing in rats with severe burn injury through miR-181c transfer [88].

### 6.3. Proliferative Phase

During the proliferative phage, fibroblasts from surrounding normal tissues migrate into the injured site. These fibroblasts produce various matrix proteins including collagen I and III to strengthen the newly formed scar tissue. MSC-exosomes affect these dermal fibroblasts to promote migration and proliferation, and produce collagen, elastin, and fibronectin: (1) human ASC-EVs or ASC-exosomes induced migration and proliferation of dermal fibroblasts or keratinocyte in vitro [234,235]; (2) human ASC-exosomes induced collagen I/III and elastin in HDFs, and they enhanced cutaneous wound healing in mice [234,235]; (3) human fetal dermal (FD)-MSC-exosomes induced the expression of collagen I/III, elastin, and fibronectin mRNAs by activating the Notch pathway through delivering Jagged 1 protein [236]; and (4) human UC-MSC-exosomes were shown to contain Wnt4 and accelerated reepithelialization of burn skin in rats [237]. The wound healing effects were inhibited when the Wnt4 expression in UC-MSC-exosomes was knock-downed by siRNA. Furthermore, human MSC-exosomes were reported to induce proliferation and migration of fibroblasts in vitro from diabetic wound patients [250]. The positive effects of MSC-exosomes on keratinocytes were also reported as follows: (1) human UC-MSC-exosomes protect the immortalized human keratinocytes HaCaT from heat-induced apoptosis by activating the AKT pathway [237]; and (2) human WJ-MSC- and iMSC-exosomes increased the secretion of collagen in HaCaT [251].

As mentioned above, angiogenesis is of importance to support the oxygen needed for the proliferation of fibroblasts or other cells in the injured site [229]. It has been also reported that MSC-exosomes induce angiogenic activity of endothelial cells. Human ASC-exosomes induced tube formation of HUVECs by delivery of miR-125a, which suppresses the expression of angiogenic inhibitor delta-like 4 (DLL4) [252]. Human BM-MSC-EVs or rat BM-MSC-exosomes were also reported to enhance angiogenesis in stroke mice [118] or in rats with renal IR injury [110], respectively. Exosomes from human endometrial MSCs were reported to increase proliferation, migration, and angiogenesis of HUVECs with increased expression levels of angiogenic markers including Tie2, angiopoietin 1 (Ang1), Ang2, and vascular endothelial growth factor (VEGF) [253]. In addition, the following pro-angiogenic effects of MSC-exosomes have been confirmed in vivo: (1) human umbilical cord blood (UCB)-MSC-exosomes with thrombin preconditioning accelerated cutaneous wound healing in rats with full-thickness wounds. Human UCB-MSC-exosomes increased the angiogenic factors such as angiogenin (Ang), Ang1, hepatocyte growth factor (HGF), and VEGF, while reducing TNF-α and IL-6 [238]; (2) human UC-MSC-exosomes enhanced angiogenesis in rats through the Wnt4/β-catenin pathway. The pro-angiogenic effects of human UC-MSC-exosomes was abolished when the Wnt4 expression was knock-downed by shRNA [239]; and (3) human iMSC-exosomes accelerated both the formation and maturation of new vessels in the wound sites with unknown mechanism [241].

### 6.4. Remodeling Phase

MSC-exosomes might be beneficial to further reduce scar formation. Uncontrolled accumulation of myofibroblasts in the wound sites causes scar formation. Recently, human UC-MSC-exosomes have been reported to reduce scar formation by inhibiting accumulation of myofibroblasts in mice [242]. A variety of proteases such as matrix metalloproteinases (MMPs) are necessary for all phases of the cutaneous wound healing process [254]. During the remodeling phase, controlled release of MMPs by fibroblasts, macrophages, epidermal cells, and endothelial cells contributes to degrading the majority of collagen III fibers [255]. Regulation of extracellular matrix remodeling by ASC-exosomes has been reported [235]. In this study, it was demonstrated that ASC-exosomes promoted scarless cutaneous wound repair by regulating the ratios of collagen I-to-collagen III, TGF-β3-to-TGF-β1, and MMP3-to-MMP1.

### 6.5. Proteolytic Environment

Uncontrolled protease activities are known to be associated with impaired wound healing [256]. Additionally, prolonged high levels of protease activities have been suggested to be associated with delayed wound healing in chronic wounds [254,255,256,257]. In fact, elevated levels and activities of collagenase (MMP-1 and MMP-8) and gelatinases (MMP-2 and MMP-9) are characteristics of chronic wounds [255]. This highly proteolytic environment is not favorable for advanced biologicals such as growth factors [258]. In fact, the use of platelet-derived growth factor (PDGF) for treatment of chronic wounds has been reported with modest effect [259]. Based on this, a clinical study for the treatment of chronic wounds with a combination of topical growth factors and proteinase inhibitors was recently initiated [260]. The proteolytic environment of chronic wounds might be also unfavorable for the treatment of MSC-exosomes, since the surface proteins on the exosomes are susceptible to proteolysis, which may change the interaction between exosomes and recipient cells [261]. Therefore, a protease-resistant formulation of MSC-exosomes would be necessary for the maximum efficacy, especially for topical applications, as reported for PDGF [262,263]. Recently, human gingival MSC (GMSC)-exosomes with chitosan/silk hydrogel showed enhanced wound healing in diabetic rats with appropriate swelling and moisture retention capacity suggested as effects of this hydrogel [242]. This hydrogel may also provide protection of exosomes from proteases in the wound site.

### 6.6. Animal Models

Most of the animal studies for wound healing with MSC-exosomes have been performed in rodents, except for two studies with rabbit and dog [232,243] (Table 5). However, the structure and physiology of rodent skin do not reflect those of human skin. Pigs are the most optimal preclinical models for wound healing because of the highest similarities between pig and human skin including skin architecture, hair density, and physiology of the wound healing process [264,265,266,267,268]. It is necessary to confirm the effects of MSC-exosomes on cutaneous wound healing in pig models for better understanding of MoA and clinical applications.

### 6.7. ASC-Exosomes

The beneficial effects of fat graft on wound repair are widely accepted, while the underlying mechanism remains unknown [269]. These effects might be related to exosomes from the subcutaneous fat layer. Recently, it has been revealed that human ASC-exosomes induce proliferation and migration of HDFs, and the expression of N-cadherin, cyclin 1, PCNA, collagen I/III, and elastin in HDFs in vitro, which results in reduced scar formation in mice by regulating extracellular matrix remodeling [234,235]. No direct evidence that shows an advantage of ASC-exosomes over exosomes from other MSCs is available. ASCs, however, are distinct in immunomodulation compared to BM-MSCs. BM-MSCs enter the wound site through the blood supply to initiate the first phase of wound healing [270]. In the injured site, BM-MSCs prolong and enhance the inflammation by increasing survival and function of neutrophils [271]. Under hypoxic conditions, which induces the activation of TRL4, BM-MSCs secreted pro-inflammatory factors and decreased the polarization of macrophage from M1 to M2 phenotype [272,273]. Therefore BM-MSCs in the wound site might not induce the anti-inflammatory M2 macrophages without enough oxygen supply by neovascularization. On the contrary, phenotype and secretome of ASCs were largely unaffected by prolonged hypoxia [274], and the CM from ASCs showed better inducing effects of the anti-inflammatory M2 macrophage phenotype than the CM from BM-MSCs [275]. These results suggest that ASC-exosomes might be more beneficial than BM-MSC-exosomes to induce appropriate wound healing processes. In summary, MSC-EVs or MSC-exosomes contribute to each phase of wound healing by inducing M2 polarization and stimulating dermal fibroblasts to produce structural proteins and proteases necessary for remodeling of the extracellular matrix.

## 7. MSC-Exosome-Induced Hair Growth

Hair follicle cycling is a dynamic and complex process involving alternating phases of rapid growth (anagen), regression (catagen), and quiescence (telogen) [276]. Hair follicles, which reside in the dermal layer of the skin, are made up of various cell types including dermal papilla (DP) cells and outer root sheath (ORS) keratinocytes, each having distinct roles [277]. In addition to these cells, ASCs located in the adipose tissue below dermis may also affect hair cycling as ASCs differentiate into mature adipocytes and surround hair follicles during the telogen to anagen transition [278]. Although a direct relationship between dermal papilla cells and ASCs has not been elucidated, it can be anticipated that ASCs exert effects on hair growth, as numerous studies have shown that transplantation of ASCs and CM from ASCs enhance proliferation of DP cells in vitro and promote hair growth in mice and human [279,280,281]. Indeed, interactions between these cell types through various mediators lead to transition from the telogen to anagen phase. Activation of the Wnt/ß-catenin signaling is one of the main pathways involved in the hair follicle development. Previous studies have shown that dermal Wnt ligands regulate hair-inducing activity of DP cells by maintaining the anagen phase [282,283]. In addition, growth factors such as fibroblast growth factor-5 (FGF-5) produced by ORS cells or insulin-like growth factor-1 (IGF-1) produced by DP cells increase proliferation of hair follicle cells [284,285]. Thus, the Wnt/ß-catenin signaling and secretion of growth factors are crucial for hair growth.

Dysregulation of hair cycling caused by various factors such as environmental, genetic, hormonal, and aging, results in hair loss [286,287,288]. Currently, finasteride and minoxidil are the mainstay treatments for alopecia, although they are not fundamental treatments that induce hair growth, not to mention having various side effects associated with them [289,290]. Hair transplantation is frequently utilized as a fundamental treatment of hair loss but it is an invasive procedure and graft survival rate largely depends on the surgeon [291]. There is a strong unmet need of a minimally invasive treatment that not only retards hair loss but also promotes hair growth.

### 7.1. The Effects of DP-Exosomes on Hair Cells

As DP cells are the key player in hair follicle cycling as they secrete growth factors, activate the Wnt signaling, and promote differentiation of hair follicle stem cells, it can be anticipated that exosomes derived from DP cells can also modulate hair follicle cycling. Indeed, studies have shown that exosomes derived from DP cells (DP-exosomes) promote hair growth. Cutaneous injection of human DP-exosomes increased the anagen to catagen ratio in mice and stimulated proliferation and ß-catenin expression of ORS cells [292]. Exosomes derived from 3D culture of human DP cells increased the percentage of Ki67-positive cells in cultured hair follicles and induced hair follicles in mice implanted with human DP spheres by activating the Wnt and bone morphogenic protein (BMP) signaling [293]. A study by Yan et al. identified 34 differentially expressed miRNAs that are involved in proliferation and differentiation of hair follicle stem cells by goat DP-exosomes [294].

### 7.2. The Effects of MSC-Exosomes on Hair Growth

Similar to DP-exosomes, MSC-exosomes are also known to carry a myriad of growth factors and Wnt activators in their cargo. For instance, human UC-MSC-exosomes were found to transport Wnt4 and Wnt11 and subsequently activate the Wnt signaling and promote cell proliferation in target cells [237,239,295]. Therefore, MSC-exosomes are attractive treatment options for hair growth as well. However, to date, there is only one publication reporting the effects of MSC-EVs on hair growth [296]. The authors showed that mouse BM-MSC-EVs promoted proliferation of human DP cells and induced secretion of growth factors such as VEGF and IGF-1, which are essential for hair growth [285,297,298]. In addition, when mice were intradermally injected with BM-MSC-EVs, an increased anagen to telogen ratio was evident in C57BL/6 mice, along with elevated Wnt protein levels in the dorsal skin. These results suggest that MSC-EVs or MSC-exosomes might have the potential to promote hair growth. Further studies will be necessary to elucidate the potential of various MSC-exosomes on hair follicle cycling.

## 8. Repair and Regeneration of Skin barrier by MSC-Exosomes

The skin is the largest organ in the human body, comprising about 15% of the total body weight, and is well known as the barrier between the external environment and the human body, preventing loss of moisture and protecting the body from UV light, pathogens, chemicals, and mechanical injuries [299]. The skin is composed of three layers: epidermis, dermis, and hypodermis. The epidermis is the outermost layer of skin and functions as the waterproof barrier. The dermis is a layer below the epidermis, consisting of tough connective tissues, hair follicles, sebaceous glands, apocrine glands, lymphatic vessels, blood vessels, and sweat glands. The hypodermis (also known as subcutaneous tissue) is the deepest layer of skin and is composed of fat and connective tissue [300,301].

### 8.1. Skin Barrier

The skin barrier is commonly divided into three distinct functional barriers: microbiome, chemical, and physical barriers [302]. The microbiome barrier comprises the outer side of the skin barrier and is composed of diverse microbial communities such as bacteria, fungi, and viruses [295]. The skin microbiome can protect the body against exogenous exposure and invasion of pathogens and can affect immune cell maturation in the skin development. It also functions as the skin immune mediator, which cross-talks between skin cells and the skin immune system [302]. In some cases, altered microbial states result in skin disease [303]. As an example, the increased abundance of *Gemella* and *Streptococcus* species are observed in AD [304].

The chemical barrier provides the acidic surface pH, which is the key factor of desquamation and regeneration of the skin barrier [303]. It also provides the lipid barrier of ceramides, cholesterol, and free fatty acids, consisting of a molar ratio of 1:1:1 [305]. The lipids prevent loss of moisture from skin and invasion of environmental substances. Furthermore, free fatty acids contribute to the homeostasis of the barrier function, maintaining acidic pH in skin [306]. In addition, the chemical barrier, especially the biochemical barrier, provides antimicrobial peptides. Antimicrobial peptides are a major factor of the innate immune systems and build the first line of defense against bacteria and viruses [307].

The physical barrier consists of stratum corneum (SC) and tight junction (TJ). The SC is the outermost layer of epidermis consisting of dead keratinocytes (corneocytes) [308]. Living keratinocytes are transformed into non-living corneocytes during cornification. Cornification is completed by the replacement of the cell membrane with a layer of ceramides covalently linked to the cornified envelope. This ceramide–corneocyte complex in SC contributes to the skin’s barrier function [309]. The epidermal TJ not only anchors cells to the neighboring cells, but also prevents the escape of moisture between cells [310]. If TJ is damaged, the Langerhans or dendritic cells, which are located below the TJ network, stretch their dendrites to the upper side of TJ, and then are activated by allergens and lead to allergic responses [301,311].

Dysfunction and damage of the skin barrier leads to several diseases such as AD [310], psoriasis [310], rosacea [312], and acne vulgaris [313]. Up to now, most of the therapeutic approaches for these diseases have targeted inflammation: (1) dupilumab, a dual inhibitor of IL-4 and IL-13, was recently approved to treat AD [314]; (2) monoclonal antibodies inhibiting IL-12, IL-23, or IL-17 are being developed for the treatment of psoriasis [315]; (3) a topical drug, ivermectin, for the treatment of mild-to-moderate rosacea has an anti-inflammatory effect [316]; and (4) anti-inflammatory drugs are also used to treat acne vulgaris, although the first line treatment of acne vulgaris is antibiotics [317]. Moisturizers, used to reduce xerosis or dryness, could prove to be toxic to individuals with compromised skin while being harmless to those with normal skin [318]. Physiologic lipid-based barrier creams, containing three essential lipids including ceramides, cholesterol, and free fatty acids, have been reported to improve barrier function and reduce pruritus as well [318]. However, currently no treatment option is available to repair or regenerate skin barrier functions.

### 8.2. The Effects of ASC-Exosomes on Skin Barrier

Recently, human ASC-exosomes have been reported to promote epidermal barrier repair in a mouse AD model [109]. Repeated exposures of oxazolone to hairless mice induced AD-like symptoms including inflammation and skin barrier abnormalities [319]. Subcutaneous injection of ASC-exosomes induced restoration of the skin barrier by production of ceramides and dihydroceramides with long acyl chains in a dose-dependent manner. ASC-exosomes also induced synthesis of sphingoids including sphingosine and S1P, increased SphK1 activity, and reduced S1P lyase (S1P1) activity in the injured skin. As mentioned previously, the S1P/Sphk1/S1PR axis is of importance for inducing M2 macrophage polarization by ASC-exosomes, which reduce inflammation and promote cutaneous wound healing [94]. Further study will be needed to elucidate the role of M2 macrophage polarization by ASC-exosomes in skin barrier repair. In addition, ASC-exosomes increased the number of epidermal lamellar bodies and formation of the lamellar layer at the interface of SC and stratum granulosum. Transcriptome analysis of diseased skins revealed that ASC-exosomes reversed the abnormal expression of genes involved in skin barrier maintenance, lipid metabolism, the cell cycle, and inflammatory responses induced by repeated oxazolone exposures. These results suggest that ASC-exosomes could be a promising cell-free treatment for the regeneration of the skin barrier in various diseases with skin barrier defects.

## 9. Application of MSC-Exosomes for Regenerative Aesthetics

Physical changes in skin over time produce psychosocial impacts that significantly affect social interactions [320]. With the global increase of older individuals over 65, there is an expanding demand for repair or rejuvenating products and procedures for aged skin [300,320]. Stem cell conditioned media (CM), mostly from the culture of MSCs, have been used as a skin care product for anti-aging, anti-wrinkle, and skin and hair care [321]. MSC-CM contain beneficial secretomes, including secreted growth factors as well as exosomes. However, MSC-CM also contain unintended ingredients including media components and additives, and cellular waste such as lactate and ammonia, both of which are restricted in cosmetics [322,323]. On the contrary, isolated MSC-exosomes avoid these potential harmful components. Currently, the tangential flow filtration (TFF) method is recommended as a suitable industrial-scale method to isolate exosomes among various techniques [40,324]. The TFF method can markedly reduce the levels of lactate and ammonia from exosome preparation (Ha et al. unpublished observation). Recently, it has been demonstrated that human ASC-exosomes isolated by the ExoSCRT™ technology, a TFF-based exosome isolation method, are safe, showing no adverse effects in GLP toxicological tests including skin sensitization, in vitro photosensitization, eye and skin irritation, or acute oral toxicity in accordance with OECD guidelines [325]. In addition, the commercial product ASCE™ (the trademark of ExoCoBio), the ASC-exosome isolated by the ExoSCRT™ technology, was firstly registered as a cosmetic ingredient in the International Cosmetic Ingredient Dictionary (ICID). The TFF-isolated ASC-exosomes have multiple effects on the skin: (1) inducing regeneration of epidermal skin barrier by increasing synthesis of ceramides, dihydroceramides, sphingosine, and S1P [110]; (2) reducing inflammation through downregulation of multiple cytokine levels [20,109,325]; (3) reducing the level of TSLP, a pruritus-causing cytokine [110]; (4) inducing synthesis of collagen and elastin in HDFs [325]; and (5) inducing proliferation of HDFs and HDPs (Ha et al. unpublished observation). Recently, a potential effect of ASC-exosomes on subcutaneous fat has been also suggested. Mouse ASC-exosomes promoted WAT beiging through induction of M2 macrophage polarization in WAT of obese mice [95]. Under the same condition, ASC-exosomes induced proliferation of ASCs themselves. Further studies are needed to decipher the effects of human ASC-exosomes on subcutaneous fat in normal physiological conditions.

**Figure 1:**
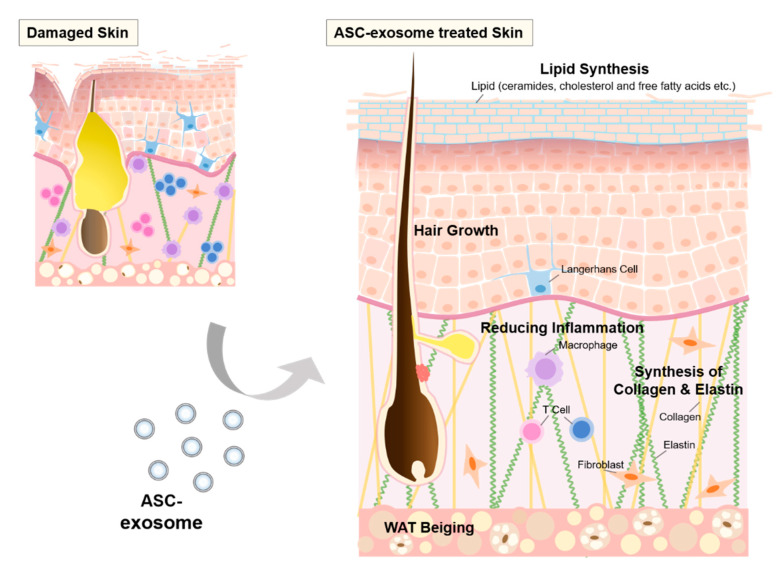
Effects of ASC-exosomes on skin.

The safety and efficacy of secretomes from different cells were analyzed for skin and wound care products, and it was found that secretome from ASCs is safer and more effective than that from BM-MSCs in many aspects: (1) lack of expression of major histocompatibility complex (MHC) class II on ASCs; (2) induction of higher levels of anti-inflammatory M2 macrophages by ASC-CM than by BM-MSC-CM; and (3) suppression of cancer growth by ASC-exosomes both in vivo and in vitro [326,327]. ASC-exosomes could be a preferable regenerative aesthetic ingredient since an important function of ASCs in skin is signaling to surrounding cells to induce the differentiation of dermal fibroblasts and keratinocytes, and activate epidermal stem cells including hair follicles [326]. A pioneering cosmeceutical product, the ASCE+™ lyophilized human ASC-exosomes (ASCE+ is the trademark of ExoCoBio), showed various beneficial effects including anti-inflammation and reduction of downtime after ablative skin treatments such as laser therapies (unpublished observation). Taken together, ASC-exosomes could be a next-generation product for the regenerative aesthetics, which affects multiple layers of skin including the epidermis (keratinocytes), dermis (fibroblast, inflammatory cells, and hair follicle), and potentially the hypodermis (subcutaneous fat) (Figure 1).

## 10. Conclusions

With the recent burst of research, MSC-exosomes are now widely accepted as next-generation cell-free therapeutics for intractable diseases. Many challenges in industrialization of exosomes are still out there such as large-scale culture of MSCs, continuous supply of MSCs with comparable therapeutic effects, and accurate determination of quantity and quality of exosomes. However, technical advances in the MSC cell therapy field, with the expected first marketing approval by the US FDA in the near future [328], are also able to be integrated in the exosome industry soon. The use of immortalized MSCs, with similar functionalities and safety profile compared to naïve MSCs, might be also an alternative strategy for stable production of MSC-exosomes [329,330]. Successful commercialization of MSC-exosomes may provide a completely new therapeutic paradigm for human healthcare.

## Figures and Tables

**Table 1:** Mesenchymal stem cell (MSC)-exosomes from different sources.

Diseases/Focuses	Nomenclature	Exosome Isolation	MSC Origin	Outcome	Reference
Alzheimer’s disease	Exosomes	Ultracentrifugation	Human adipose tissue	Adipose stem cell (ASC)-exosomes had superior effects compared to bone marrow (BM)-MSC-exosomes Decreased Aβ peptide in the N2a cells	[30]
			Human bone marrow		
Glioblastoma	Extracellular Vesicles (EVs)	Ultrafiltration	Human bone marrow	Decreased U87MG cell proliferation Induced apoptosis in the U87MG cells	[31]
			Human Wharton’s jelly		
			Human adipose tissue	Increased U87MG cell proliferation No apoptotic effect	
Neurodegenerative disease	Exosomes	Ultracentrifugation	Human menstrual fluid	Promoted neurite outgrowth in cortical and sensory neurons	[32]
			Human bone marrow		
			Human chorion	No effect	
			Human umbilical cord		
Osteoarthritis (OA)	Exosomes	Ultrafiltration	Human iPSCs	Attenuated OA in a murine model Stimulated chondrocyte migration and proliferation Induced pluripotent stem cell-derived MSC (iMSC)-exosomes exert superior therapeutic effects compared to synovial membrane (SM)-MSC-exosomes	[33]
			Human synovial membrane		
Exosome release	Exosomes	Ultracentrifugation	Canine bone marrow	BM-MSCs released higher amount of exosome compared to ASCs	[34]
			Canine adipose tissue		
	Exosomes	Total Exosome Isolation Kit (Invitrogen)	Human amniotic fluid	Amniotic fluid (AF)-MSCs released higher amount of exosome compared to BM-MSCs	[35]
			Human bone marrow		

**Abbreviations:** AF, amniotic fluid; ASC, adipose stem cell; BM, bone marrow; EVs, extracellular vesicles; iMSC, induced pluripotent stem cell-derived MSC; OA, osteoarthritis.

**Table 2:** Quality control (QC) criteria in the guidelines and good manufacturing practice (GMP) settings.

QC Criteria	Examples in Guidelines		Examples in GMP Settings		
	ISEV Recommendation [43,44,45]	MFDS Guideline (2018) [46]	Pachler et al. [47]	Andriolo et al. [48]	Mendt et al. [42]
Exosome Quantity	Particle number by NTA, high-resolution FCM RPS, cryo-EM, AFM, etc.	Particle number by NTA or compatible methods ^1^	(ZetaVeiw NTA)	(NanoSight NTA)	(NanoSight NTA)
	Total protein amount	^2^	-	(BCA assay)	(microBCA assay)
	Total lipid amount	-	-	-	-
	Total RNA amount	-	-	-	-
	Quantification of specific molecules	-	-	TSG101 ELISA	-
Exosome Size	NTA	NTA ^1^			
	-	DLS ^1^	-	-	-
	RPS	RPS ^1^	-	-	-
	High-resolution FCM	-	-	-	-
	AF4	-	-	-	-
		-	-	-	-
	FCS	FCS ^1^	-	-	-
Identity	Proteins	Proteins	(WB: CD9, CD81, TSG101)	(FCM: CD9, CD63, CD81 ELISA: TSG101)	(FCM: CD47, CD63, CD81, CD9, CD29, CD90)
	Phospholipids	Lipids	-	-	-
	Nucleic acids	RNAs	-	-	-
Purity	Ratio of protein:particle	-	-	-	-
	Ratio of lipids:particle	-	-	-	-
	Ratio of lipids:protein	-	-	-	-
	Proteins that are expected not to be enriched in exosomes	Proteins that are expected not to be enriched in exosomes	(WB: GM130)	-	-
	Process impurities depending on the source of exosomes	Process impurities (serum albumin, antibiotics, etc.)	-	-	-
Potency Assays	Dose-response assessment	Biological assay, which can represent MoA	-	Anti-apoptotic activity; Pro-angiogenic activity	Apoptosis assay
Others	Not mentioned	Mycoplasma test	-	-	-
		Sterility test	-	Microbiological Control for Cellular Products	-
		Endotoxin test	-	Quantitative LAL test	-
		Adventitious virus test	-	-	-

^1^ Since these methods cannot differentiate EVs from non-EV particles, it is recommended to compare results from these methods with results from TEM, AFM, or other microscopic observation. ^2^ Comparison with results from quantification methods such as protein quantification is also recommended. **Abbreviations**: AF4, multi-angle light scattering coupled to asymmetric flow field-flow fractionation; AFM, atomic force microscopy; DLS, dynamic light scattering; FCM, flow cytometry; FCS, florescence correlation spectroscopy; ISEV, International Society for Extracellular Vesicles; LAL, limulus amebocyte lysate; MoA, mode of action; MFDS, Ministry of Food and Drug Safety; NTA, nanoparticle tracking analysis; RPS, resistive pulse sensing; WB, Western blotting.

**Table 3:** Anti-inflammatory and immunomodulatory effects of MSC-exosomes.

Category	Exosome Source	Nomenclature	Exosome Isolation	Related Exosomal Cargo	Secreted Factors or Expressed Genes Affected	Immunomodulatory Effects	Reference
Macrophage polarization	Human jaw bone marrow (JM-MSCs) Human BM-MSCs	Exosomes	Ultracentrifugation ExoQuick (System Biosciences)	miR-223	TNF-α ↓ IL-10 ↑	Accelerated wound healing in mice Induced M2 macrophage polarization (CD206+ macrophage ↑)	[85]
	Human JM-MSCs Human BM-MSCs	Exosomes	Ultracentrifugation	-	Collagen, *Il-6*, *Ccl2*, *Cd206*, *Ccl7*, *Ccl17*, *Tnfα*, *Retnia* ↓ *Arg1* ↑	Reduced BPD through macrophage M22 polarization	[86]
	Human umbilical cord (UC)-MSCs	Exosomes	Ultracentrifugation	let-7b	TLR4, p-p65, iNOS ↓ p-STAT3, p-AKT, ARG1 ↑	Alleviated inflammation and enhanced diabetic cutaneous wound healing in rats Induced M2 macrophage polarization Inhibited TLR4 signaling pathway	[87]
	Human UC-MSCs	Exosomes	PureExo (101Bio)	miR-181c	TNF-α, IL-1β, TLR4, p65, p-p65 ↓ IL-10 ↑	Reduced burn-induced inflammation in rats Reduced neutrophil and macrophage infiltration (MPO+ cell, CD68+ cell ↓) Inhibited TLR4 signaling pathway	[88]
	Human menstrual blood derived MSCs (MenSCs)	Exosomes	Ultracentrifugation	-	iNOS ↓ ARG1, VEGF ↑	Resolved inflammation and ameliorate cutaneous non-healing wounds in diabetic mice Induced M2 macrophage polarization	[89]
	Mouse BM-MSCs	Exosomes	HPLC	let-7	HMGA2, IGF2BP1 ↓	Attenuated atherosclerosis in mice Reduced area of atherosclerotic plaques Promoted M2 macrophage polarization	[90]
	Mouse BM-MSCs	Exosomes	Ultracentrifugation	miR-182	IL-6, iNOS, IL-1 β, IL-6, TNF-α ↓ ARG1, IL-10, TGF-β ↑	Reduced myocardial ischemic-reperfusion injury in mice Reduced infarct size and inflammation Promoted M2 macrophage polarization	[91]
	Human BM-MSCs	Exosomes	Ultracentrifugation	MT2A	IFN-γ, IL-1β, IL-6, TNF-α ↓ IL-10, *Lyz1*, *Defa20*, *Defa29*, *Ang4* ↑	Reduced IBD by polarizing M2 macrophage in mice	[92]
	Rat ASCs	Exosomes	Ultracentrifugation	-	S1P, SphK1, S1PR1 ↑ AGR1, Ym1, TGF-β1, IL-10 ↑ IL-1β, IL-6, TNF-α, IFN-γ, p65 ↓	Reduced cardiac damage in rats Reduced fibrosis and apoptosis Promoted M2 macrophage polarization	[93]
	Human ASCs	Exosomes	Exosome Isolation Kit (System Biosciences)	-	CD163, ARG1, CD206, STAT6, MafB ↑	Increased the expression of M2 macrophage markers	[94]
	Mouse ASCs	Exosomes	Ultrafiltration	STAT3	ARG1, IL-10, tyrosine hydroxylase ↑ TNF-α, IL-12 ↓	Induced M2 macrophage polarization in obese mice ASC-exosome-educated M2 macrophage promoted WAT beiging	[95]
T cell regulation	Human BM-MSCs	Exosomes	ExoQuick (System Biosciences)	-	TNF-α, IL-1β ↓ TGF-β ↑	Induced conversion of Th1 into Th2 Reduced differentiation of Th17 Increased the level of Tregs Induced apoptosis of PBMCs and CD3+ T cells	[96]
	Human BM-MSCs	Exosomes	Ultracentrifugation	-	IL-10, TGF-β ↑	Promoted proliferation and immune-suppression capacity of Tregs	[97]
	Human UC-MSCs	Exosomes	PEG6000 precipitation	-	IL-10, IDO ↑	Induced an increase of Tregs in PBMCs Inhibited proliferation of PBMCs	[98]
	Human embryonic stem cell (ES)-MSCs	Exosomes	Tangential flow filtration + HPLC	EDA-FN	TNF-α, IL-1β, IL-6, IL-12p40 ↓ IL-10 ↑	Induced Tregs through activation of APCs in the MyD88-dependent manner Enhanced allogeneic skin graft	[99]
	Mouse ASCs	Exosomes	Ultracentrifugation	-	IL-17, IFN-γ ↓ IL-4, IL-10, TGF-β ↑	Ameliorated autoimmune type 1 diabetes mellitus by increasing Tregs in mice	[100]
	Human BM-MSCs	Exosomes	Ultracentrifugation	-	IL-6, IL-12p70, IL-22, IL-17AF ↓ IDO ↑	Improved motor skill in the MS mouse experimental autoimmune encephalomyelitis model Increased Tregs and decreased infiltration and proliferation of pro-inflammatory T cells	[101]
	Mouse BM-MSCs	Exosomes	Ultracentrifugation	-	IL-1, IL-2, IL-4, IL-10, TNF-α, IFN-γ ↓	Decreased aminotransferase (ALT), liver necrotic areas, and apoptosis in Con A-induced liver injury in mice Increased Tregs	[102]
	UC-MSCs	EVs	Size exclusion chromatography	-	-	Suppressed T cell proliferation	[105]
B cell regulation	Human BM-MSCs	Exosomes	Ultracentrifugation	-	MZB1, CXCL8 ↑ IgM ↓	Reduced proliferation of T and B cells	[106]
Photoaging	Human BM-MSCs	Exosomes	Ultrafiltration	-	TNF-α, IL-1β ↓ TGF-β, CTLA4 ↑	Reduced photoaging of skin in mice Ameliorated inflammation	[107]
Skin flap	Human ASCs	Exosomes	Ultracentrifugation	-	-	Enhanced neovascularization and survival of the skin flap in rats Reduced inflammation and apoptosis	[108]
Atopic dermatitis (AD)	Human ASCs	Exosomes	Tangential flow filtration	-	IgE, IL-4, IL-5, IL-13, IL-17, IL-23, IL-31, IFN-γ, TNF-α, TSLP ↓	Reduced pathological symptoms of AD in mice Reduced mast cell infiltration Reduced inflammatory dendritic epidermal cells (CD86+/CD206+ cells↓)	[20,109]
Renal injury	Rat BM-MSCs	Exosomes	Ultracentrifugation	-	MDA, HIF1α, NOX2, Caspase 3, BAX, PARP1, MPO, ICAM1, IL-1β, NF-κB ↓ SOD, CAT, GPX, HO-1, BCL2, IL-10, bFGF, HGF, SOX9, VEGF ↑	Decreased histopathological score of kidney injury in rats Reduced the levels of blood urea nitrogen (BUN) and creatinine Reduced the level of oxidative stress Increased anti-oxidant status Reduced apoptosis and inflammation Improved regeneration and enhanced angiogenesis	[110]
	Mouse BM-MSCs	Exosomes	Ultracentrifugation	CCR2	TNF-α, IL-6, IL-1β ↓	Reduced BUN and creatinine in the mouse IR model Reduced infiltration of macrophages	[111]
	Human UC-MSCs	Exosomes	Ultracentrifugation	-	PCNA, BCL-XL, BCL2, IL-1β, 4E-BP1 ↑ Bax, cytochrome C, Caspase-3, p65, TNF-α, IL-6, IL-1β, p-mTOR ↓-	Reduced cisplatin-induced AKI in rats Reduced BUN and creatinine	[112]
Uveitis	Human UC-MSCs	Exosomes	Ultracentrifugation	-	-	Reduced experimental autoimmune uveitis in rats Reduced infiltration of Gr-1+, CD161+, CD68+ and CD4+ cells in retina	[113]
Duchenne muscular dystrophy (DMD)	Human Placenta MSCs	Exosomes	Ultracentrifugation	miR-29c	TGF-β, creatine kinase, collagen I, collagen IV, TNF-α, IL-6 ↓ Utrophin ↑	Reduced DMD in mice Decreased the tissue fibrosis and inflammation	[114]
Bronchopulmonary dysplasia (BPD)	Human UC-MSCs	Exosomes	Ultracentrifugation	TSG-6	Neutrophil ↓	Improved pathology of lung, cardiac and brain in neonatal mice with BPD Reduced pulmonary inflammation and alveolar-capillary leak	[115]
Alzheimer’s disease	Mouse BM-MSCs	Exosomes	Ultracentrifugation	-	TNF-α, IL-1β, IL-6 ↓ IL-10, IL-4, IL-13 ↑	Improved cognitive function in transgenic APP/PS1 mice Reduced plaque deposition and Aβ levels Reduced activation of astrocytes	[116]
Post-stroke neuroregeneration	Human BM-MSCs	EVs	PEG6000 precipitation	-	Dcx, NeuN, CD31 ↑	Improved neurological impairment (motor coordination) and long-term neuroprotection (neuronal survival and cell proliferation) in stroke mice Reduced post-ischemic immunosuppression and lymphopenia Stimulated post-ischemic neurogenesis and angiogenesis	[117]
Diabetic peripheral neuropathy	Mouse BM-MSCs	Exosomes	Ultracentrifugation	miR-17 miR-23a miR-125b	TNF-α, IL-1β, iNOS, TLR4, IRAK1, p65 ↓ ARG1, IL-10, TGF-β ↑	Decreased the threshold for thermal and mechanical stimuli in mice Increased nerve conduction velocity, the number of intraepidermal nerve fibers, myelin thickness, and axonal diameters	[118]
OA	Rabbit BM-MSCs	Exosomes	Ultracentrifugation	-	p-p38, p-ERK ↓ p-AKT ↑	Increased chondrocytes viability under IL-1β-induced inflammatory status through activating AKT pathway	[119]
	Human ES-MSCs	Exosomes	Tangential flow filtration	CD73	α-SMA, MMP-13, IL-1β, iNOS ↓ PCNA, s-GAG ↑	Promoted repair and regeneration of temporomandibular joint OA in rats through the AKT/ERK/AMPK-dependent manner	[120]
	Human BM-MSCs	Exosomes	ExoQuick (System Biosciences)	miR-26a-5p	PTGS, Bcl-2, IL-6, TNF-α, IL-8, IL-1β ↓ Bax, caspase-3 ↑	Alleviated OA damage in rats treated with pentobarbital	[121]
	Human ES-MSCs	Exosomes	Tangential flow filtration	CD73	TNF-α, IL-1β ↓ PCNA ↑	Induced cartilage repair through the CD73-mediated activation of AKT and ERK pathway	[122]
Intervertebral disc degeneration (IVDD)	Mouse BM-MSCs	Exosomes	Ultrafiltration	-	Caspase-9/3, iNOS, MMP-3/13, caspase-1, IL-1β, TXNIP, NLRP3 ↓ COL2A, SOX9 ↑	Prevented progression of IVDD in rabbit Suppressed activation of NLRP3 inflammasome	[123]
Spinal cord injury	Human UC-MSCs	EVs	Ultracentrifugation		IL-1β, IL-6 ↓	Demonstrated anti-inflammatory and anti-scarring activities in the spinal cord parenchyma in rats	[124]
	Rat BM-MSCs	Exosomes	Ultracentrifugation	-	C3, GFAP, TNF-α, IL-1α, IL-1β, p-p65, p-IκBα ↓	Reduced spinal cord injury-induced A1 astrocytes in rats	[125]
	BM-MSCs	Exosomes	Ultrafiltration	-	NO, Bax, caspase-3, TNF-α, IL-1β, IL-6 ↓ Bcl2, VEGF, NF200 ↑	Improved functional behavioral recovery in rats Attenuated neuronal cells apoptosis, suppressed glial scar formation Suppressed activation of microglia, A1 neurotoxic reactive astrocytes and neuroinflammation	[126]
Myocardial infarction	Rat BM-MSCs	Exosomes	Total Exosome Isolation Kit (Invitrogen)	miR-29, miR-24	-	Inhibited cardiac fibrosis, inflammation, and improved cardiac function in rat myocardial infarction model	[127]
	Rat BM-MSCs	Exosomes	ExoQuick (System Biosciences)	-	NO, Bax, caspase-3/9 ↓ Bcl2 ↑	Improved microenvironment of infarcted myocardium in rats through angiogenesis and anti-inflammation	[128]
Acute lung injury (ALI)	Rat BM-MSCs	Exosomes	Exosome extractant (Ribobio Co., Ltd.)	miR-124-3p	P2X7, TNF-α, IL-6, IL-8 ↓ GSH, SOD ↑	Increased survival rate of rats	[129]
	Rat BM-MSCs	Exosomes	Ultracentrifugation		TNF-α, IL-1β, IL-6, MMP-9 ↓ IL-10, SP-C ↑	Attenuated phosgene-induced ALI in rats	[130]
	Rat BM-MSCs	Exosomes	Ultracentrifugation	-	Caspase-3, TNF-α, IL-1β, IL-6, TLR4, NF-κB ↓	Attenuated ischemia repurfusion (IR)-induced lung injury in rats Decreased apoptosis and inflammation	[131]
Induced pulmonary fibrosis (IPF)	Human BM-MSCs	Exosomes	Ultracentrifugation	-	CCL2, ARG1 ↓	Reduce bleomycin-induced IPF in mice Reduced collagen deposition and apoptosis	[132]
Hepatic IR injury	Human iMSCs	Exosomes	Ultrafiltration		TNF-α, IL-6, HMGB1, caspase-3, Bax ↓ GSH, GSH-Px, SOD ↑	Suppressed hepatocyte necrosis and sinusoidal congestion Reduced the AST and ALT	[133]
Liver fibrosis	Human UC-MSCs	Exosomes	Ultrafiltration	-	AST ↑ Collagen I/III, TGF-β 1, p-Smad2 ↓	Alleviated hepatic inflammation and collagen deposition in the CCl_4_-induced fibrotic liver of mice	[134]
Acute liver failure	Mouse ASCs	Exosomes	Total Exosome Isolation Kit (Invitrogen)	miR-17	TNF-α, IFN-γ, IL-1β, IL-6, IL-18, TXNIP, NLRP3, ASC, caspase-1 ↓	Ameliorated acute liver failure by reducing ALT and AST in mice Reduced activation of TXNIP/NLRP3 inflammasome in macrophages	[135]
Intestinal bowel disease (IBD)	Human UC-MSCs	Exosomes	Ultracentrifugation	-	TNF-α, IFN-γ, IL-1β, IL-6, IL-17 ↓ TGF-β 1, IL-10 ↑	Ameliorated DSS-induced IBD in mice	[136]
Necrotizing enterocolitis (NEC)	Mouse BM-MSCs	Exosomes	PureExo (101Bio)	-	-	Reduced incidence and severity of NEC in premature newborn rats	[137]
Abdominal aortic aneurysm	Human UC-MSCs	EVs	Ultracentrifugation	miR-147	IL-6, IL-17, IFN-γ, IL-23, RANTES, KC, MCP-1, MIP-1α, HMGB1 ↓	Reduced inflammation and macrophage activation in a mouse abdominal aortic aneurysm model	[138]
Perinatal brain injury	Human Wharton’s jelly (WJ)-MSCs	Exosomes	Ultracentrifugation	-	TNF-α, IL-6, IL-1β, CXCL10, IκBα, p-ERK1/2, p-JNK, p-p38 ↓	Reduced neuroinflammation in rats with perinatal brain injury	[139]
	Human WJ-MSCs	Exosomes	Ultracentrifugation	-	Mbp, Map 2 ↑	Reduced neuron-specific cell death in rats with perinatal brain injury	[140]
Traumatic brain injury (TBI)	Rat BM-MSCs	Exosomes	ExoQuick (System Biosciences)	-	GFAP ↑	Improved spatial learning in rats with TBI	[141]
	Human ASCs	Exosomes	ExoQuick (System Biosciences)	MALAT1	TNF-α, IL-1β, IFN-γ ↓	Improved motor behavior in rats with TBI	[142]
Hypoxic-ischemic brain injury	Human BM-MSCs	EVs	PEG6000 precipitation	-	-	Improved function of brain by reducing the total number and duration of seizures in sheep	[143]
Urethral stricture	Human UC-MSCs	Exosomes	Ultracentrifugation	miR-146a	α-SMA, collagen I/III, IL-6, IL-1β, IRAK1, TRAF6, NF-κB ↓	Reduced urethral fibrosis and stricture in rats	[144]
Status epilepticus (SE)	Human BM-MSCs	Exosomes	Anion exchange chromatography	-	TNF-α, IL-1β, MCP-1, SCF, MIP-1a, GM-CSF ↓ IL-10, PDGF-B, IL-6, IL-2 ↑	Reduced pilocarpine-induced SE in mice Reduced loss of glutamatergic and GABAergic neurons Reduced inflammation in hippocampus	[145]
	Human UC-MScs	Exosomes	Ultracentrifugation	-	GFAP, TNF-α, IL-1β ↓	Ameliorated SE-induced learning and memory impairment in mice	[146]
Retinal IR injury	Human BM-MSCs	EVs	ExoQuick (System Biosciences)	-	TNF-α, IL-6, caspase-3 ↓	Reduced neuro-inflammation and apoptosis	[147]
Laser-induced retinal injury	Mouse ASCs Human UC-MSCs	Exosomes	Ultracentrifugation		MCP-1 ↓	Reduced damage, inhibited apoptosis, and suppressed inflammatory responses in mice	[148]
Sepsis	Mouse BM-MSCs	Exosomes	Ultracentrifugation	miR-223	TNF-α, IL-1β, IL-6 ↓	Protected cardiomyocytes from cecal ligation and puncture-induced sepsis in mice through downregulation of SEMA3A and STAT3	[149]
Graft versus Host Disease (GvHD)	Human UC-MSCs	EVs	Ultracentrifugation	-	IL-2, TNF-α, IFN-γ ↓ IL-10 ↑	Prevented acute GvHD in a mouse model of allogeneic hematopoietic stem cell transplantation	[150]
	Human BM-MSCs	Exosomes	PEG6000 precipitation	-	TNF-α, IL-1β, IFN-γ ↓	Modulated the patient’s immune cells	[151]

Abbreviations: AD, atopic dermatitis; ALI, acute lung injury; BPD, bronchopulmonary dysplasia; DMD, Duchenne muscular dystrophy; ES, embryonic stem cell; IBD, intestinal bowel disease; IPF, induced pulmonary fibrosis; IR, ischemia reperfusion; IVDD, intervertebral disc degeneration; JM, jaw bone marrow; MenSCs, menstrual blood derived MSCs; SE, status epilepticus; UC, umbilical cord; WJ, Wharton’s jelly.

**Table 4:** Anti-senescence effects of exosomes derived from stem cells.

Exosome Source	Nomenclature	Exosome Isolation	Potential MoA	Senescent Cells	In Vitro Effects	In Vivo Effects	Reference
Human ASCs	Exosomes	ExoQuick (System Biosciences)	NFR2	HG-induced senescent EPCs	Cell viability, Tube formation ↑ SMP30, p-VEGFR2 ↑ NOX1, NOX4, IL-6, IL-1β, TNF-α ↓	Wound healing in diabetic rat	[205]
Human UC-MSCs	Exosomes	Total exosome isolation kit (Invitrogen)	Reducing NF-κB/TNFα signaling by lncRNA MALAT1	H_2_O_2_-treated H9C2	SA-β-gal ↓ NF-κB activation, p21, TNFα ↓ Cell proliferation ↑	Improvement cardiac function in D-gal-induced aged mouse	[206]
Human UC-MSCs	Exosome	Ultracentrifugation	TGF-β1 downregulation by miR-675	H_2_O_2_-treated H9C2	SA-β-gal, p21, TGF-β1 ↓	Perfusion in ischemic hindlimb	[207]
Human BM-MSCs Human iPSCs	EVs	Size exclusion chromatography	Reduction of ROS by PRDXs enriched in exosomes	RS MSCs Progerin-induced senescent MSCs	Cell growth ↑ SA-β-gal, IL-1A, IL-6, γ-H2AX↓ ↓ p21, p53 mRNAs ↓	ND	[208]
Human ASCs	Exosomes	Ultracentrifugation	Unknown	IL-1β-treated OA osteoblasts	SA-β-gal, γ-H2AX ↓ IL-6 and Prostaglandin E2 ↓ Oxidative stress, Mitochondrial membrane potential ↓	ND	[209]
Rat BM-MSCs	Exosomes	Ultracentrifugation	Activation of Wnt/β-catenin signaling	Irradiated rat BM-MSCs	Oxidative stress ↓ γ-H2AX, Rb, p53, p21, p16 ↓ SOD1/2, Catalase ↑	Attenuating radiation-induced bone loss in rat	[210]
Mouse ESCs	Exosome	ExoQuick (System Biosciences) or Ultracentrifugation	TGF-β Receptor 2 inhibition by mouse miR-291a-3p (human miR-371a-3p	RS HDFs AS HDFs	SA-β-gal ↓ Cell proliferation, migration ↑	ND	[211]
Human ESCs	Exosome	Ultracentrifugation	KEAP1 downregulation by miR-200a	D-gal-induced HUVECs	SA-β-gal, p16, p21 ↓ ROS ↓ Cell proliferation, migration, tube formation ↑	Pressure ulcer healing in D-gal-induced aged mouse	[212]
Human iPSCs	Exosomes	ExoQuick (System Biosciences)	Unknown	RS HDFs Photoaged HDFs	SA-β-gal, MMP-1/3 ↓ Collagen Type I ↑	ND	[213]
Human iPSCs	Exosomes	Ultracentrifugation	Unknown	HG-injured HUVECs	SA-β-gal ↓Cell viability, Tube formation↑	ND	[214]

**Abbreviations**: AS, adriamycin-induced cellular senescence; HG, high glucose; ND, not determined; IRS, ionizing radiation-induced senescence; RS, replicative senescence.

**Table 5:** Wound healing effects of MSC-exosomes.

Exosome Source	Nomenclature	Exosome Isolation	Related Exosomal Cargo	Factors Affected	Animal for In Vivo Study	Reference
Human JM-MSCs Human BM-MSCs	Exosomes	Ultracentrifugation ExoQuick (System Biosciences)	miR-223	TNF-α ↓ IL-10 ↑	Mouse	[85]
Human UC-MSCs	Exosomes	Ultracentrifugation	let-7b	TLR4, p-p65, iNOS ↓ p-STAT3, p-AKT, ARG1 ↑	Rat	[87]
Human UC-MSCs	Exosomes	PureExo (101Bio)	miR-181c	TNF-α, IL-1β, TLR4, p65, p-p65 ↓ IL-10 ↑	Rat	[88]
Human ASCs	Exosomes	ExoQuick (System Biosciences)	-	NOX1, NOX4, IL-6, IL-1β, TNF-α ↓ SMP30, p-VEGFR2 ↑	Rat	[205]
Rabbit ASCs Rabbit BM-MSCs	EVs	Ultracentrifugation	-	-	Rabbit	[232]
Human ASCs	EVs	Ultracentrifugation	-	-	Rat	[233]
Human ASCs	Exosomes	ExoQuick (System Biosciences)	-	N-cadherin, cyclin 1, PCNA, collagen I/III, elastin ↑	Mouse	[234]
Human ASCs	Exosomes	ExoQuick (System Biosciences)	-	Collagen I/II, TGF-β1/3, MMP1/3 α-SMA ↓	Mouse	[235]
Human fetal dermal MSCs	Exosomes	ExoQuick (System Biosciences)	Jagged 1	Collagen I/III, elastin, fibronectin mRNA ↑	Mouse	[236]
Human UC-MSCs	Exosomes	Ultracentrifugation	Wnt4	CK19, PCNA, collagen I ↑	Rat	[237]
Human UC blood-MSCs	Exosomes	Ultracentrifugation	-	Ang, Ang1, HFG, VEGF ↑	Rat	[238]
Human UC-MSCs	Exosomes	Ultracentrifugation	Wnt4	β-catenin, N-cadherin, PCNA, Cyclin D3 ↑	Rat	[239]
Human iPSC-MSCs	Exosomes	Ultracentrifugation	-	Collagen I/III, elastin, ↑	Rat	[240]
Human UC-MSCs	Exosomes	Ultracentrifugation	-	α-SMA, collagen I ↓	Mouse	[241]
Human gingival MSCs	Exosomes	Size exclusion chromatography	-	Collagen ↑	Rat	[242]
Dog BM-MSCs	Exosomes	Ultracentrifugation	-	α-SMA ↓	Dog	[243]
